# Bayesian models to explain autistic traits in psychiatric population

**DOI:** 10.1192/j.eurpsy.2021.642

**Published:** 2021-08-13

**Authors:** M. Benassi, S. Garofalo, L. Vitali, M. Orsoni, R. Sant’Angelo, R. Raggini, G. Piraccini

**Affiliations:** 1 Department Of Psychology, University of Bologna, Cesena, Italy; 2 Mental Health Department, AUSL ROMAGNA, Cesena, Italy

**Keywords:** executive function, Bayesian model, autistic traits, Psychiatric symptoms

## Abstract

**Introduction:**

Studies on psychiatric patients have shown that the presence of autistic traits affects the effectiveness of the treatment, decreasing the likelihood of positive clinical outcomes.

**Objectives:**

The aim of the present study is to investigate which are the areas of overlap between psychiatric symptoms and the traits of the autism spectrum using a bayesian approach.

**Methods:**

A sample of 190 adult psychiatric patients, diagnosed with schizophrenia, bipolar disorder, major depression, and personality disorder participated in the study. The RAADS-R questionnaire was used to assess the presence of autistic traits. The severity of psychiatric symptoms was measured with the BPRS and PANSS scales, the perceived well-being and disability using the Whodas and Whoqol scales, the TOL and STROOP for the measurement of executive functions, the attentional matrices for visual-spatial attention, the Raven for general cognitive skills.

**Results:**

No difference emerged between the diagnoses regarding the presence of symptoms of the autism spectrum, which affects 64% of subjects. Logistic regression showed that the severity of symptoms measured as BPRS and PANSS predicted the probability of having autistic traits. Bayesian regression showed that specific autistic traits are indicative of executive functions deficits. Namely, motor impairment severity measured at RAADS is strongly predicted by rule violation with number of correct moves measured at TOL. The other executive functions seemed to be only moderately linked to autistic traits.

**Conclusions:**

These results provide new information about the expression of comorbidity with autism in psychiatric patients.

